# Cranial Autonomic Symptoms in Migraine Patient Presenting in the Department of Neurology of a Tertiary Care Center: A Descriptive Cross-sectional Study

**DOI:** 10.31729/jnma.8576

**Published:** 2024-05-31

**Authors:** Parash Rayamajhi, Pravesh Bhattarai, Janak Khadka, Sujit Khanal, Subodh Chapagain

**Affiliations:** 1 Department of Neurology, Kathmandu Medical College and Teaching Hospital, Kathmandu, Nepal; 2 Kathmandu Medical College and Teaching Hospital, Kathmandu, Nepal; 3 Dhuliknel Hospital, Kavre, Nepal

**Keywords:** *cranial autonomic symptoms*, *migraine*, *trigeminal autonomic cephalagias*

## Abstract

**Introduction:**

Cranial autonomic symptoms are typically associated with trigeminal autonomic cephalagias and are also a part of TACs' diagnostic criteria. However, they have also been commonly reported in migraine patients. This study aimed to find the prevalence of crania! autonomic symptoms in migraine patients who presented to the Department of Neurology in a tertiary care center.

**Methods:**

This descriptive cross-sectional study was conducted among migraine patients who visited the Department of Neurology of a tertiary care center from September 2023 to December 2023 after obtaining ethical approval from the Institutional Review Committee. Neurologists used ICHD-3 beta criteria to diagnose migraine and the presence of cranial autonomic symptoms in patients with migraine through face-to-face interviews using a structured questionnaire. A convenience sampling method was used. The point estimate was calculated at a 95% Confidence Interval.

**Results:**

Among 119 migraine patients, at least one cranial autonomic symptom was seen in 76 (63.86%) (55.23-72.51, at 95% Confidence Interval). Lacrimation 34 (44.73%) and conjunctival injection 27 (35.52%) were the two most commonly reported symptoms. Bilateral CAS was present in 60 (78.94%) patients.

**Conclusions:**

The prevalence of at least one CAS in migraine patients was found to be similar to other studies done in similar settings.

## INTRODUCTION

Cranial Autonomic Symptoms (CAS) in migraine include lacrimation, conjunctival injection, eyelid edema, nasal congestion, rhinorrhea, miosis, ptosis, forehead or facial sweating, and facial flushing.^1^ Activation of the trigeminal autonomic reflex (anatomic brainstem connection between the trigeminal cervical complex and cranial parasympathetic outflow system) is known to cause CAS in migraine.^2^ CAS are commonly missed to be diagnosed as these symptoms are not mentioned in the diagnostic criteria of migraine.

There is a high prevalence of migraine in the Nepali community.^3^ A population-based study in Nepal revealed that the prevalence of migraine in Nepal is very much higher than the mean global estimate of 14.7 %.^3^ While a substantial body of research exists for CAS in migraine patients in Caucasian and Japanese populations, a gap exists for a similar understanding of CAS in the Nepalese population.

This study aimed to find the prevalence of CAS in migraine patients who presented to the neurology OPD of a tertiary care hospital.

## METHODS

A descriptive cross-sectional study was conducted on migraine patients who visited the Department of Neurology of Kathmandu Medical College and Teaching Hospital, Sinamangal, Kathmandu, Nepal, from September 2023 to December 2023. The ethical approval was taken from the Institutional Review Committee of the same institute (Reference number: 20092023/03). Patients of all age groups who gave consent and who were diagnosed with migraine by an expert neurologist according to ICHD-3 (Beta version) criteria were included.^4^ Patients who were diagnosed with migraine were provided with structured questionnaires of CAS using the criteria of ICHD-3 beta. The study excluded patients who denied to give consent, have evidence of comorbidity, or diabetes mellitus, and who cannot provide reliable history. Convenience sampling was used. The sample size was calculated using the formula.


$$\begin{array}{ll} \text{n} & ={\text{Z}}^{2}\times \frac{\text{p}\times \text{q}}{{\text{e}}^{\text{2}}}\\ & =1.96^{2}\times \frac{0.74\times 0.26}{0.10^{2}}\\ & =74 \end{array}$$

n= minimum required sample size
z= 1.96 at 95% Confidence Interval (CI)
p= prevalence taken as 74% from the previous study^5^
q= 1-p
e= margin of error, 10%

The calculated sample size was 74. However, 119 patients were included in our study.

Information was collected from the patients from face-to-face interviews. Demographic profiles such as age and sex were recorded from all the patients. A structured questionnaire about CAS symptoms was asked for the migraine patients using ICHD 3 beta criteria, namely 1) lacrimation, 2) conjunctival injection, 3) aural fullness, 4) eyelid edema, 5) nasal congestion, 6) Sweating in face and forehead, 7) facial flushing, 8) rhinorrhea, 9) ptosis,10) miosis.^1^ Patients were also asked about the laterality of CAS in relation to headache i.e. bilateral, unilateral.

Data were entered and analyzed using Statistical Package for the Social Sciences( SPSS). The point estimate was calculated at a 95% Confidence interval.

## RESULTS

Among 119 patients with migraine, at least one cranial autonomic symptom was seen in 76 (63.86%) (55.23-72.51 at 95% CI). The mean age of migraine patients with CAS positive was 34.36±12.87. Age-wise distribution reported that higher proportions, 44 (57.89%) of CAS-positive migraine patients belonged to the age group of 21-40 years, and the lowest proportion, 2 (2.63%), fall on ages greater than 60 years. More than three-fourths i.e. 62 (81.57%) of CAS-positive migraine patients were reported in females (Table 1).

**Table 1:** Demographic characteristics (n= 119).

Age group (years)	n (%)
≤20	15 (12.60)
21-40	73 (61.34)
41-60	29 (24.36)
> 60	2 (1.70)
**Sex**	
Female	99 (83.20)
Male	20 (16.80)

Among 76 CAS-positive patients, 53 (69.73%) had no more than two symptoms (Table 2).

**Table 2:** Number of cranial autonomic symptoms among CAS-positive patients (n= 76).

Number of CAS	n (%)
One CAS	29 (38.15)
Two CAS	24 (31.58)
Three CAS	12 (15.79)
Four or more CAS	11 (14.47)

According to our study, lacrimation 34 (44.73%), conjunctival injection 27 (35.52%), and aural fullness 24 (31.57%) were the three common cranial autonomic symptoms seen in migraine patients. Ptosis and miosis are least commonly seen in 5 (6.57%) and 1 (1.31%) respectively (Table 3).

**Table 3:** Frequency of cranial autonomic symptoms in Migraine patients (n= 76).

Cranial autonomic symptoms	n (%)
Lacrimation	34 (44.73)
Conjunctival injection	27 (35.52)
Aural fullness	24 (31.57)
Eyelid edema	23 (30.26)
Nasal congestion	20 (26.31)
Sweating on face and forehead	19 (25.00)
Facial flushing	12 (15.78)
Rhinorrhea	8 ( 10.52)
Ptosis	5 (6.57)
Miosis	1 (1.31)

Bilateral CAS was present in 60 (78.94%) patients among which 49 had a bilateral headache and 11 had unilateral headaches (Table 4). Unilateral CAS was reported in 16 (21.05%) among which 5 had bilateral headaches and 11 had unilateral headache.

**Table 4:** Laterality of cranial autonomic symptoms in relation to headache in migraine patients (n= 76).

Laterality	n (%)
Bilateral	60 (78.94%)
Unilateral	16 (21.05%)

## DISCUSSION

The prevalence of at least one CAS in migraine patients was 76 (63.9%) in our study. Our study finding corresponds with the results of the previous study, which have a prevalence of 60.95%.^6^ Additionally, our results were also similar to those of another population-based study.^7^ The prevalence rate of CAS in migraine varies enormously in literature ranging from 27% to 82%.^8^ This might be probably due to variations in the study population and study methodology. The highest prevalence, 82%, was reported in a study conducted among chronic migraine patients, and a study with low prevalence was conducted in the general population.^7^ The study in Iran also depicted that at least one CAS is present in 70% of chronic migraine and 56.2% of episodic migraine.^6^

In the present study, lacrimation is seen as the most prevalent CAS. A previous study also reported a similar prevalence, with a lacrimation of 44.2% in CAS-positive migraine patients.^9^ However, some studies demonstrated a higher prevalence of this ocular CAS.^10,11^ As lacrimation is easily perceptible and observable in a patient with pain, this may be the reason for the symptom being commonly reported.

Nasal symptoms, i.e., rhinorrhoea and nasal congestion, were also reported in our study. A study done in Taiwan had the prevalence of nasal symptoms to be 35%, which is similar to our study.^9^ However, the study done in Germany reported a lower prevalence.^12^ This may be because only unilateral nasal symptoms were taken into account in the later study, contrary to us, where we have taken both bilateral & unilateral symptoms.

Aural fullness accounted for 24 (31.57%) of CAS in our study, which is slightly higher than the study done in India.^5^ The findings in our study suggest that aural fullness is also widely present in CAS. They also support the inclusion of aural fullness in ICHD 3 beta.

Aural symptoms may develop as the trigeminal nerve also innervates the cochlear nucleus and superior olivary complex.

The current study demonstrated 60 (78.94%) of patients had bilateral symptoms & 16 (21.05%) had unilateral symptoms. This is similar to a study done in India. One study reported that bilateral CAS in unilateral migraine may be due to the crossing of fibers in the brain stem.^8^ Also, CAS is more common in bilateral migraineurs because of diminished or absent reciprocal inhibition between two locus coerulei.^13^ Males accounted for 18.42% of CAS & females accounted for 81.47% of CAS in our study. This is similar to a study done in the UK.^14^ This may be because the female gender is reported to influence central sensitization positively.^13^

This study was interview-based, so recall bias cannot be ruled out. As this study was conducted with a small sample size and selected from a population from our single tertiary center, the findings may not be representative of a broader population. Our study also did not report the clinical characteristics of migraine, in patients with and without CAS.

## CONCLUSIONS

The prevalence of CAS in migraine patients is similar to other studies done in similar settings. It suggests higher chronic migraine patients & patients with severe pain. There are overlapping symptoms of migraine with CAS & TAC, so proper history, through clinical examination, is required for accurate diagnosis and treatment. Further future prospective clinical studies are needed on CAS comparing migraine and cluster headaches. Additional awareness must be established about CAS in migraine.
